# The Intracellular Localisation and Phosphorylation Profile of the Human 5-Lipoxygenase Δ13 Isoform Differs from That of Its Full Length Counterpart

**DOI:** 10.1371/journal.pone.0132607

**Published:** 2015-07-14

**Authors:** Eric P. Allain, Luc H. Boudreau, Nicolas Flamand, Marc E. Surette

**Affiliations:** 1 Département de Chimie et Biochimie, Université de Moncton, Moncton, Canada; 2 Centre de recherche de l’Institut universitaire de cardiologie et de pneumologie de Québec, Département de médecine et Faculté de médecine, Université Laval, Québec, Canada; University of Alabama at Birmingham, UNITED STATES

## Abstract

5-Lipoxygenase (5-LO) catalyzes leukotriene (LT) biosynthesis by a mechanism that involves interactions with 5-lipoxygenase activating protein (FLAP) and coactosin-like protein (CLP). 5-LO splice variants were recently identified in human myeloid and lymphoid cells, including the catalytically inactive ∆13 isoform (5-LO∆13) whose transcript lacks exon 13. 5-LO∆13 inhibits 5-LO product biosynthesis when co-expressed with active full length 5-LO (5-LO1). The objective of this study was to investigate potential mechanisms by which 5-LO∆13 interferes with 5-LO product biosynthesis in transfected HEK293 cells. When co-expressed with 5-LO1, 5-LO∆13 inhibited LT but not 5-hydroxyeicosatetraenoic acid (5-HETE) biosynthesis. This inhibition was independent of 5-LO∆13—FLAP interactions since it occurred in cells expressing FLAP or not. In cell-free assays CLP enhances 5-LO activity through interactions with tryptophan-102 of 5-LO. In the current study, the requirement for W102 was extended to whole cells, as cells expressing the 5-LO1-W102A mutant produced little 5-LO products. W102A mutants of 5-LO∆13 inhibited 5-LO product biosynthesis as effectively as 5-LO∆13 suggesting that inhibition is independent of interactions with CLP. Confocal microscopy showed that 5-LO1 was primarily in the nucleoplasm whereas W102A mutants showed a diffuse cellular expression. Despite the retention of known nuclear localisation sequences, 5-LO∆13 was cytosolic and concentrated in ER-rich perinuclear regions where its effect on LT biosynthesis may occur. W102A mutants of 5-LO∆13 showed the same pattern. Consistent with subcellular distribution patterns, 5-LO∆13 was hyper-phosphorylated on S523 and S273 compared to 5-LO1. Together, these results reveal a role for W102 in nuclear targeting of 5-LO1 suggesting that interactions with CLP are required for nuclear localization of 5-LO1, and are an initial characterisation of the 5-LO∆13 isoform whose inhibition of LT biosynthesis appears independent of interactions with CLP and FLAP. Better knowledge of the regulation and properties of alternative 5-LO isoforms will contribute to understanding the complex regulation of LT biosynthesis.

## Introduction

5-Lipoxygenase (5-LO) catalyses the initial steps of the conversion of arachidonic acid (AA) to leukotrienes (LTs), lipid mediators that play a crucial role in the inflammatory response [1]. While LTs are active participants in host defence, excessive levels of these bioactive lipids have long been linked to diseases with an inflammatory component such as asthma, atherosclerosis and inflammatory arthritis [2–7]. A better understanding of the mechanisms of control of 5-LO activation and of LT biosynthesis could therefore uncover new therapeutic approaches to the treatment of these diseases.

5-LO is mainly expressed by leukocytes. The enzyme is localized in the cytoplasm and/or the nucleoplasm of resting cells, and translocates to peri-nuclear membranes upon cell stimulation [8]. For instance, 5-LO is intra-nuclear in alveolar macrophages [9], rat basophilic leukemia cells [10] and bone-marrow derived mast cells [11], while human neutrophils have mostly cytosolic 5-LO [12]. Numerous factors are involved in the translocation and activation of 5-LO, notably arachidonic acid [13], ATP [14] andFFfffff, calcium ions [15, 16]. In addition, 5-LO also interacts with coactosin-like protein (CLP) [17], which also participates in 5-LO translocation [18]. In cell-free experiments, the tryptophan residue 102 (W102) in the N-terminal domain of 5-LO was shown to be responsible for the interaction of 5-LO with CLP, and for the CLP-induced increase in 5-LO activity in cell-free assays [19]. CLP also interacts with F-actin [20] suggesting that the cytoskeleton has a role to play in 5-LO translocation. Upon cell stimulation and subsequent binding to CLP, 5-LO translocates to the nuclear envelope where it interacts with the five-lipoxygenase-activating protein (FLAP). This interaction has yet to be fully characterized but is important for LT biosynthesis and the stable translocation to the nuclear membrane [13, 18, 21, 22] where 5-LO dimerization may also be associated with its activation [23, 24].

**Fig 1:**
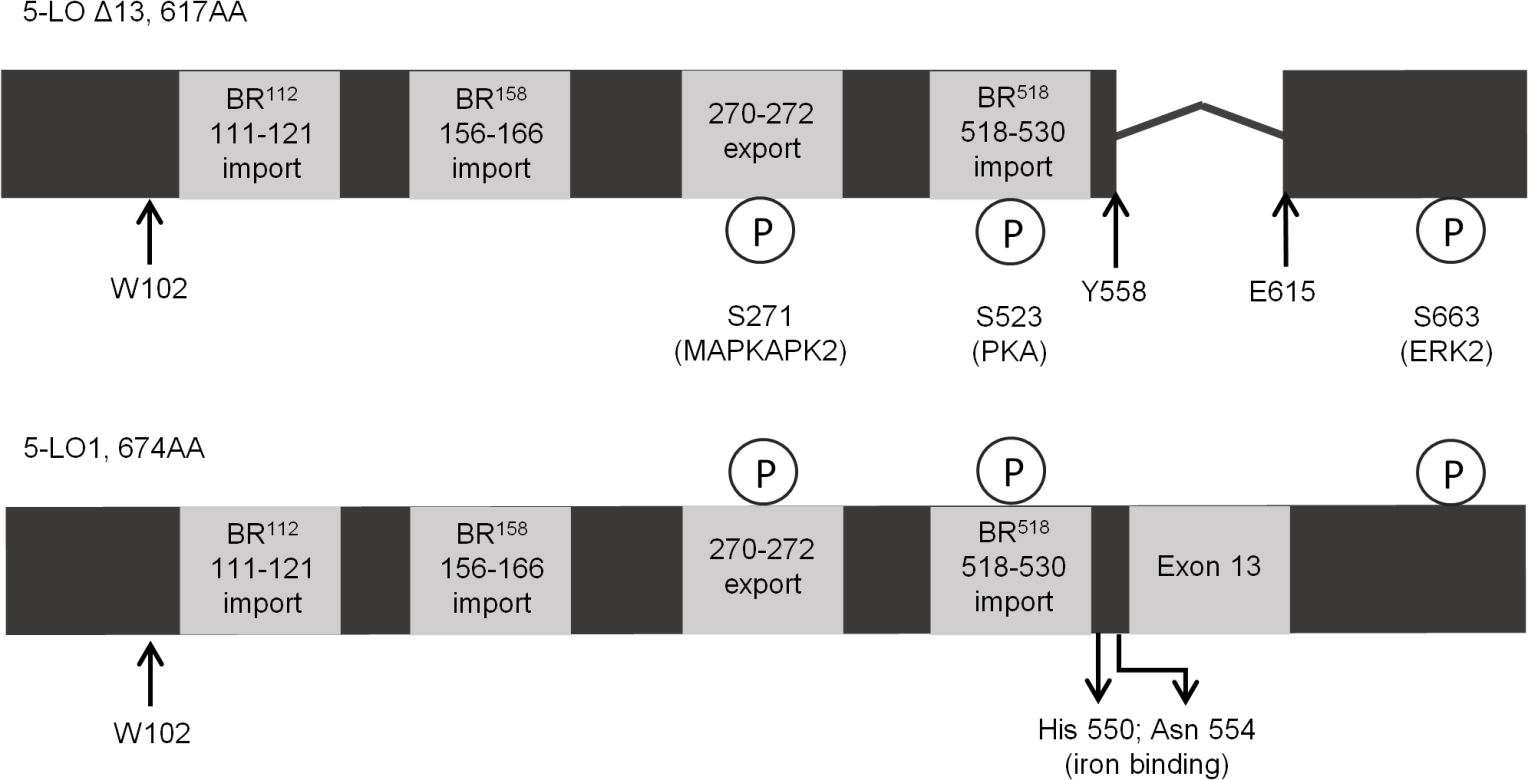
Known regulatory factors are retained in the protein sequence of 5-LO∆13. chematic of the protein sequences of the 617 amino acid 5-LO∆13 isoform (top) and of the 674 amino acid 5-LO1 isoform (bottom). All known phosphorylation sites, import and export sequences are shown to be present maintained in 5-LO∆13 despite the lack of exon 13. The kinases (MAPKAPK2, PKA and ERK) responsible for phosphorylation of the different sites are indicated as is residue W102 that is responsible for the interaction of 5-LO1 with CLP, and for the CLP-induced increase in 5-LO1 activity in cell-free assays.

The gene that codes for 5-LO, ALOX5, was suggested to be part of a multitranscript family in a study on human brain tumors where malignancy was positively correlated with 5-LO transcript abundance and multiple transcripts were observed [25]. More recently, we and others described the presence of alternatively spliced variants of 5-LO in several human cell lines and showed that at least one of these splice variants, the Δ13 isoform, is expressed in both B-lymphocyte derived cell lines and in human neutrophils [26, 27]. The Δ13 isoform protein is catalytically inactive due to the excision of exon 13 which encodes an important section of the catalytic domain [28] (Fig 1). Although the known alternative isoforms are without catalytic activity, some splice variants, including the Δ13 isoform, interfere with LT biosynthesis when co-expressed with the active 5-LO in HEK293 cells [26]. The mechanism by which alternative 5-LO protein isoforms affect LTs biosynthesis is unknown, however, a better understanding of the mechanisms by which they interfere with LT biosynthesis may provide new clues regarding the control of 5-LO activation in both healthy and diseased states.

Given the recent identification of several alternatively spliced isoforms of 5-LO, a nomenclature has been adopted for clarity. The full length catalytically active protein coded by all 14 exons of the ALOX5 gene will be designated 5-LO1 (Fig 1). The alternative isoforms will be named according to the alternative splicing event. For example the Δ13 isoform that lacks exon 13 is termed 5-LOΔ13, while the α10 isoform where intron 10 is retained is termed 5-LOα10.

An analysis of the protein sequence of the 5-LOΔ13 shows that all known regulatory factors usually housed within the active 5-LO1 are retained despite the lack of amino acids coded by exon 13 (Fig 1). The N-terminal domain is retained which is essential for calcium binding, the interaction with CLP via W102, and translocation to the nucleus upon cell stimulation. LT biosynthesis is also regulated by phosphorylation of the 5-LO enzyme. Multiple phosphorylation sites have been identified some of which are housed within classic nuclear import/export sequences that regulate 5-LO activation and localization [29]. Phosphorylation on serine 271 is associated with nuclear import and increased LT production [30], while phosphorylation on serine 523 is associated with reduced LT biosynthesis and exclusion from the nucleus [31]. Accordingly, the regions identified as basic region (BR)^518^, BR^112^ and BR^158^ [32] that regulate nuclear import of 5-LO1, as well as the nuclear export region associated with amino acids 270 to 272 [30] are also retained in 5-LOΔ13 (Fig 1). These nuclear import/export sites are highly relevant since Ser-523 lies within the BR^518^ import region and Ser-271 lies within the nuclear export region.

In this study, we further characterize 5-LOΔ13 by demonstrating that it is regulated differently than the 5-LO1 enzyme. We show here for the first time that the two proteins reside in different sub-cellular compartments, are differentially phosphorylated and possess different capacities to translocate following cell stimulation. Importantly, the inhibitory effect of 5-LOΔ13 on LT biosynthesis is independent of interactions with FLAP or with CLP.

## Methods

### Plasmids and site-directed mutagenesis

pcDNA3.1 expression vectors for 5-LO1 and 5-LO∆13 and a pBUDCE4.1 vector expressing FLAP-hemagglutinin (FLAP-HA) were prepared as previously described [26]. W102A mutants were generated by directed mutagenesis of 5-LO1 and 5-LO∆13 constructs using the QuickChange Lightning Site-Directed mutagenesis kit according to the manufacturer’s protocol (Agilent Technologies). New constructs were then transformed into MAX efficiency DH5α competent cells (Life Technologies), cells were plated and colonies were selected and grown overnight in 3 ml of LB broth containing 100 μg/ml of ampicillin. Alkaline lysis preparations for each vector were done the next day. Tubes were centrifuged at 600×g for 10 min and pellets were resuspended in 100 μL of a buffer containing 25 mM Tris, 10mM EDTA, 50mM glucose and 20 μg/mL RNase A. Lysozyme (20 μL) was then added at a concentration of 10mg/mL and tubes were incubated for 2 min before adding 200 μL of a second buffer containing 1% SDS and 200 mM NaOH. Samples were then put on ice for 5 min before adding a third buffer containing 3 M potassium (KOAc) and 5 M acetate (HAc). Tubes were then mixed by inverting, placed on ice 5 min and centrifuged at 21,000×g. Supernatants were conserved and 400 μL of phenol:chloroform was added. The upper phase was transferred to a new tube and 1 mL 99% ethanol was added before incubating for 2 min. Samples were then centrifuged for 5 min in a cold centrifuge and pellets were left to dry completely before resuspending in TE buffer. DNA constructs were then sent to the Plateforme de séquencage et génotypage du CHUL (Québec, QC) for sequencing to confirm base pair change. Colonies expressing proper sequences were incubated overnight in 200 mL LB broth containing 100 μg/mL ampicilin. Purification was then carried out using the PureLink HiPure Plasmid FP Maxiprep kit according to the manufacturer’s protocol.

### Transfections of HEK293 cells

Transfections were completed by detaching and resuspending HEK293 cells (ATCC) at a concentration of 1.5×10^7^ cells/mL. Cells were then transferred to a 400 μL electroporation cuvette (Bio-Rad), the indicated plasmids (37.5 μgDNA/ml) were added and the solutions were incubated at room temperature for 10 minutes. Cells were then shocked (250 volts, 950 μF) using a Gene Pulser Xcell (Bio-Rad) and left to sediment for 10 minutes and were then transferred to pre-warmed culture flasks containing DMEM medium supplemented with 10% foetal bovine serum (FBS) at 37°C. Experiments with transient transfections were carried out within 24–48 hours. Stable transfectants were obtained by culturing cells in DMEM medium supplemented with 10% FBS at 37°C in a humidified 5% CO_2_ environment in the presence of 400 ng/mL geneticin (Life Technologies) for pcDNA3.1 vectors or 200 ng/mL zeocin (Invivogen) for pBUDCE4.1 vectors.

### Immunofluorescence microscopy

Glass cover slides were washed with 70% ethanol and placed at the bottom of six-well plates (CellStar). Cells were centrifuged and re-suspended in DMEM containing 10% FBS at a concentration of 3×10^5^ cells/mL. One mL was then added to each six-well plate and incubated overnight. Wells were then washed with HBSS and stimulation was initiated by adding 1 ml of HBSS solution containing 1.6 mM CaCl_2_, 1 μM calcium ionophore A23187 (Sigma-Aldrich) and 10 μM arachidonic acid (AA) (Nu-Check Prep). After 10 minutes at 37°C, the glass slides were rinsed with PBS and cells were fixed with 4% paraformaldehyde (Alfa Aesar). The plates were incubated at room temperature for 20 min, rinsed with PBS and permeabilized with 0.25% Triton X-100 (Sigma-Aldrich). Following permeabilisation, the slides were rinsed with PBS containing 10% FBS for 15 minutes. Afterwards, 80 μL of rabbit anti-5-LO (1:50 in PBS with 10% FBS, Cell Signaling Technology) were placed on a sheet of parafilm and glass slides were turned over onto the drops and incubated for 1 h at room temperature. Slides were then rinsed twice for 2 min with PBS and incubated for 1 h with 1 mL of Alexa Fluor 488-conjugated goat anti-rabbit and Alexa Fluor 568-conjugated goat anti-mouse (1:800; Life Technologies). Slides were rinsed again and incubated 5 min with 4',6-diamidino-2-phenylindole (DAPI) (100 ng/mL) before finally rinsing with water and mounting slides with PermaFluor aqueous mounting medium (Thermo Scientific). Confocal laser scanning microscopy was performed with an IX81 motorized microscope equipped with a FV1000 scanning head and an Olympus 60X oil objective. Images are 200 μm cuts from samples taken by scanning at 450 nm or 488 nm. Data were acquired and exported using Fluoview 10-ASW software.

### Cell stimulation and analysis of 5-LO products

Cells were stimulated as previously described with slight modifications [26]. Briefly, HEK293 cells were detached by trypsinization and re-suspended in HBSS containing 1.6 mM CaCl_2_, 1 μM ionophore A23187 and 10 μM AA at a concentration of 1×10^7^ cells/mL and were incubated at 37°C in a water bath for 30 min. Stimulations were stopped by adding 0.5 volumes of a methanol:acetonitrile (1:1) solution containing 100 ng/mL each of prostaglandin B_2_ (PGB_2_; Cayman Chemical) and 19-OH-PGB_2_ as internal standards, and samples were then kept at -20°C overnight for protein denaturation. Samples were then centrifuged at 12,000×g for 5 min, supernatants were collected and subjected to automated in-line solid phase extraction on Oasis HLB columns prior to reverse-phase high-performance liquid chromatography analysis with diode array detection [33].

### SDS-PAGE and Western blots

Cells were trypsinized and centrifuged, then re-suspended in a lysis buffer (150 mM NaCl, 2 mM EDTA and 50 mM Tris-HCl, pH 7.6) containing 0.1% NP-40, complete mini-EDTA free protease inhibitor tablets (Roche) and phosphatase inhibitors (Sigma-Aldrich). Laemmli solution (5X, Sigma-Aldrich) was then added to a final concentration of 1X and were heated for 10 minutes in a boiling water bath.

SDS-PAGE was carried out on a 4–12% acrylamide gel gradient before transferring proteins onto a polyvinylidene fluoride membrane (GE Healthcare). Western blotting was done using rabbit monoclonal anti-5-LO (1:500 in TBS-Tween; Cell Signaling Technology), rabbit monoclonal anti-hemagglutinin (1:2000 in TBS-Tween; Cell Signaling Technology), rabbit anti-phospho-S523 5-LO (1:500 in TBS-Tween; Cell Signalling Technology), rabbit anti-phospho-S271 5-LO (1:500 in TBS-Tween; Cell Signalling Technology), and a horseradish-conjugated mouse anti-rabbit IgG (Jackson Immunoresearch). Membranes were then developed using ECL prime western blotting detection reagent (GE Healthcare) and detection was performed using an Alpha Innotech Fluorchem imager.

### Statistical analyses

Student’s t-tests and one-way ANOVA were performed to determine differences in 5-LO product biosynthesis. All values are means ± SEM. Statistics were done using Graphpad Prism version 5.

## Results

### 5-LO∆13 affects LT but not 5-hydroxyeicosatetraenoic acid (5-HETE) biosynthesis

**Fig 2:**
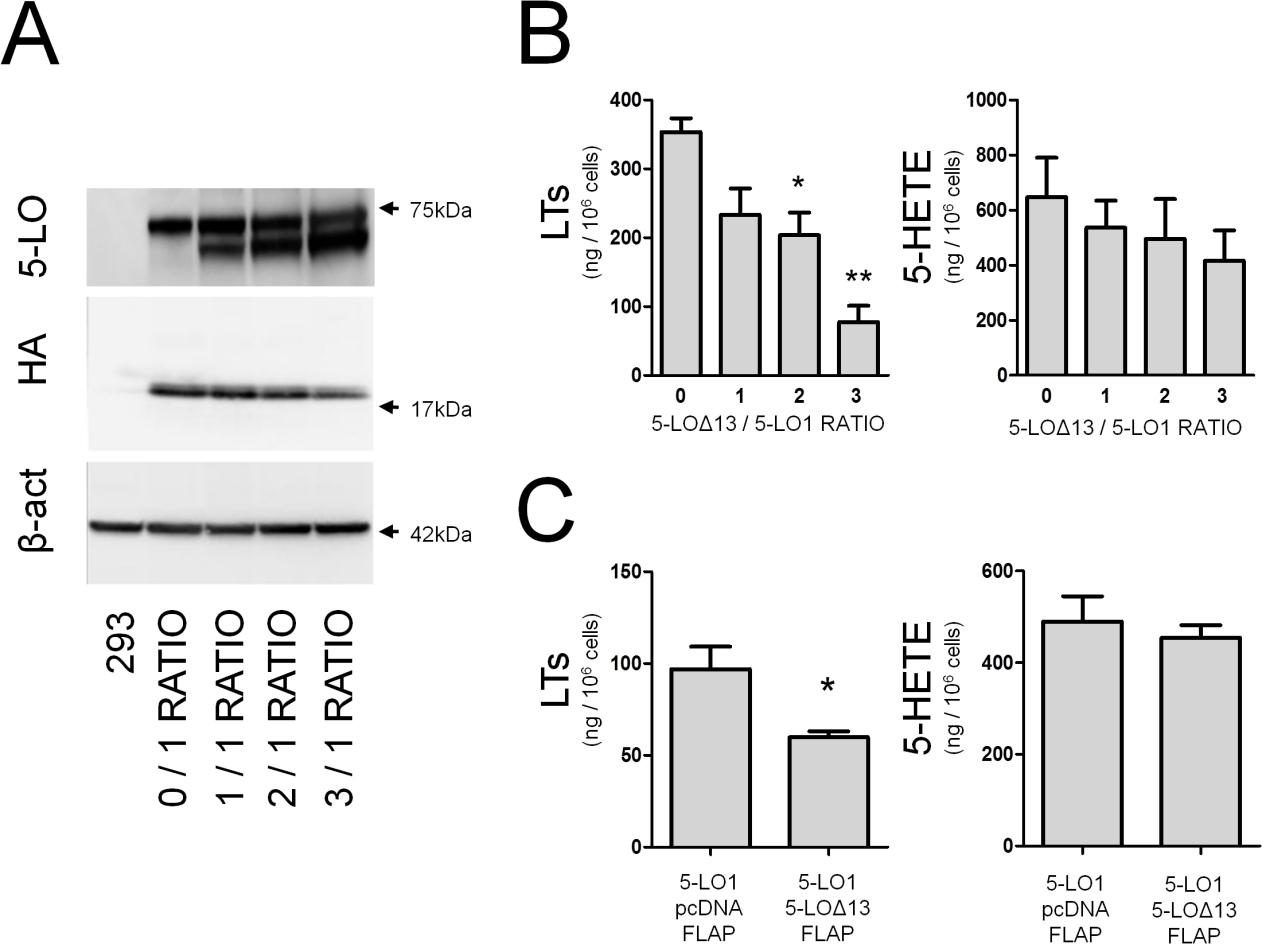
The Δ-13 isoform of 5-lipoxygenase inhibits LT biosynthesis in a dose-dependant manner. **(A)** Immunoblots showing expression of 5-LO1 (top band) and 5-LOΔ13 (lower band) after transfections with the indicated ratios of 5-LOΔ13/5-LO1 expression vectors, as well as the presence of FLAP-HA and β-actin as loading control in HEK293 cells. **(B)** HEK293 cells expressing FLAP-HA and transfected with the indicated ratios of 5-LO1 and 5-LO∆13 expression vectors were stimulated with 1 μM thapsigargin and 10 μM AA for 30 minutes. 5-LO products were measured by HPLC as described in the Methods section. Leukotrienes (LTs) are the sum of LTB_4_ and its trans isomers. 5-HETE = 5-hydroxyeicosatetraenoic acid. **(C)** HeLa cells transfected with a 1:1 ratio of vectors expressing 5-LO1 and 5-LO∆13 were stimulated under the same conditions as in (B) and LTs and 5-HETE production were measured. Immunoblots are representative of 4 independent experiments. Data represent means ± SEM of 4 independent experiments. *Different from control p<0.05, and **p<0.01.

We previously reported that 5-LO∆13 could inhibit the biosynthesis of 5-LO products when co-expressed with the active 5-LO1 [26]. To further investigate this process, we performed experiments in which we modulated the expression of 5-LO∆13 while keeping that of 5-LO1 constant. This was achieved by manipulating vector ratios in transfection experiments (Fig 2A). Increasing the amount of 5-LO∆13 decreased 5-LO product biosynthesis by 5-LO1-transfected HEK293 cells (Fig 2B). The stimulated biosynthesis of LTs was indeed diminished in a dose dependent manner and although we observed a decrease in 5-HETE production, this did not reach statistical significance. To verify whether this was unique to the HEK293 cell model, the same co-expression experiments were performed in HeLa cells and yielded similar results (Fig 2C).

### W102 is required for 5-LO product biosynthesis in intact stimulated cells

**Fig 3:**
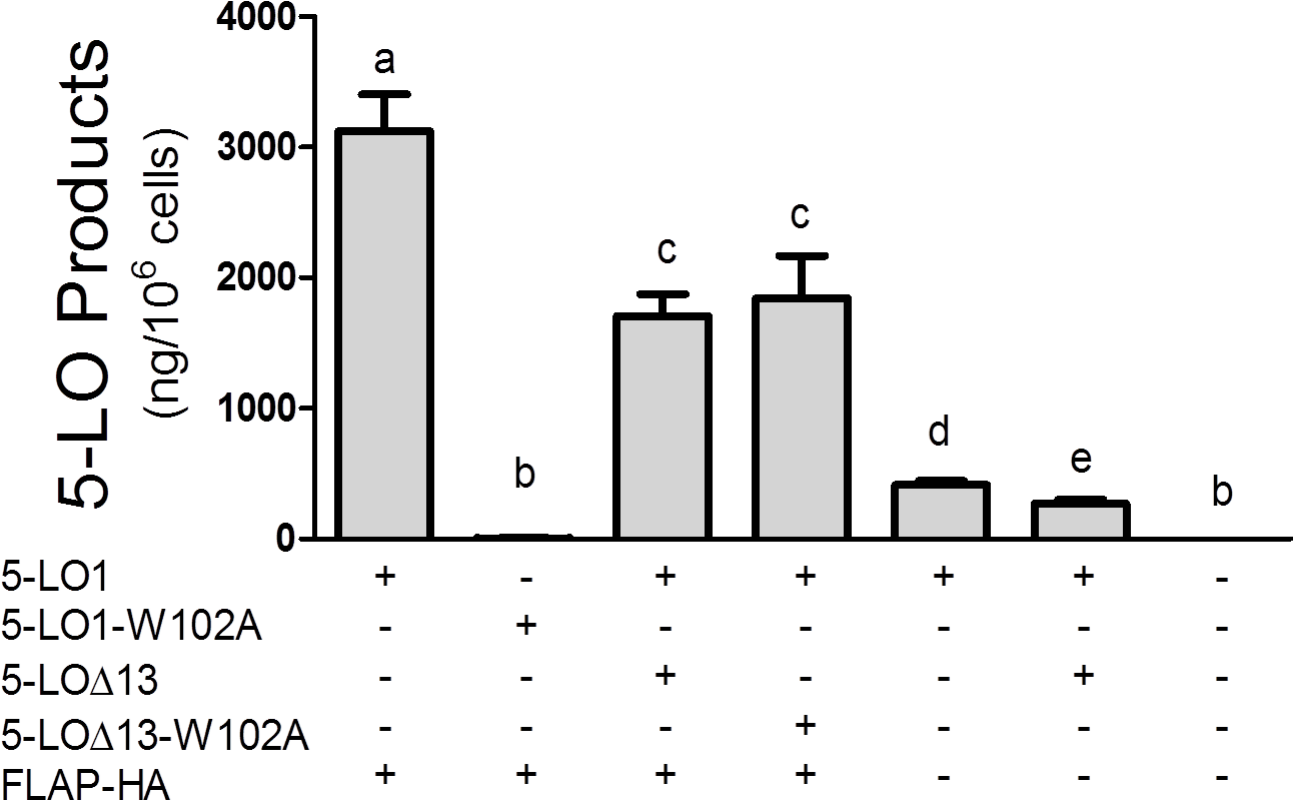
Impact of W102A mutations and of FLAP expression on the biosynthesis of 5-LO products. HEK293 cells expressing FLAP-HA or not were transfected with expression vectors coding for 5-LO1, the 5-LO1-W102A mutant, 5-LO∆13 or the 5-LO∆13-W102A mutant as shown. Control cells (last column) were transfected with a control pcDNA3.1 vector. HEK293 cells were then stimulated with 1 μM thapsigargin and 10 μM AA. 5-LO products were measured by HPLC as described in the Methods section and represent the sum of LTB_4_, its trans isomers and 5-hydroxyeicosatetraenoic acid. Data represent means ± SEM of 3 or 4 independent experiments. *Values without a common superscript are different, p<0.05.

Given that interactions of 5-LO1 with CLP greatly increase the capacity to synthesize LTs, the possibility that 5-LO∆13 inhibits 5-LO product biosynthesis by a mechanism related to CLP was next considered. A single amino acid (W102) on 5-LO1 has been shown to be directly involved in the interaction of 5-LO1 with CLP [19]. However, the role of W102 for the stimulated biosynthesis of 5-LO products in intact cells had not previously been assessed. When the W102A mutant of 5-LO1 (5-LO1-W102A) was expressed in HEK293 cells, the stimulated biosynthesis of 5-LO products was drastically reduced compared to that of 5-LO1 indicating that W102 is required for product biosynthesis, and suggesting that the interaction of CLP with the W102 of 5-LO1 is required for proper 5-LO1 activation and product biosynthesis in intact HEK293 cells (Fig 3, second column).

### Modulation of LT biosynthesis by 5-LO∆13 does not involve interactions with CLP or FLAP

Having confirmed the importance of W102 in intact cells, a W102A mutant of the catalytically inactive 5-LO∆13 (5-LO∆13-W102A) was generated to investigate whether the 5-LO∆13 protein might inhibit LT biosynthesis by trapping CLP. Co-expression experiments of 5-LO∆13-W102A with 5-LO1 showed a similar inhibition profile for the biosynthesis of 5-LO products to that of its non-mutant counterpart indicating that the inhibitory effect of 5-LO∆13 does not involve the binding and sequestration of CLP (Fig 3, third and fourth columns).

In addition to CLP, interactions of the 5-LO1 enzyme with FLAP are necessary for the efficient production of LTs in stimulated cells [34]. To evaluate the possibility that 5-LO∆13 may inhibit the biosynthesis of 5-LO products by interfering with 5-LO1/FLAP interactions, the inhibitory effect of 5-LO∆13 was assessed in HEK293 cells co-transfected with a vector expressing recombinant FLAP or a mock vector (HEK293 cells do not naturally express FLAP [26, 35]). 5-LO1 and FLAP-HA expression were confirmed by western blotting (not shown) and, as expected, cells expressing 5-LO1 stimulated in the absence of FLAP generated significantly less 5-LO products than cells co-expressing FLAP (Fig 3, compare 5^th^ column with 1^st^ column). However, 5-LO∆13 still effectively inhibited the biosynthesis of 5-LO products in the absence of FLAP suggesting that the impact of 5-LO∆13 on 5-LO product biosynthesis is independent of the presence of FLAP (Fig 3, 5^th^ and 6^th^ columns).

### Confocal microscopy evaluation of the subcellular distribution of 5-LO isoforms and their W102A mutants

**Fig 4:**
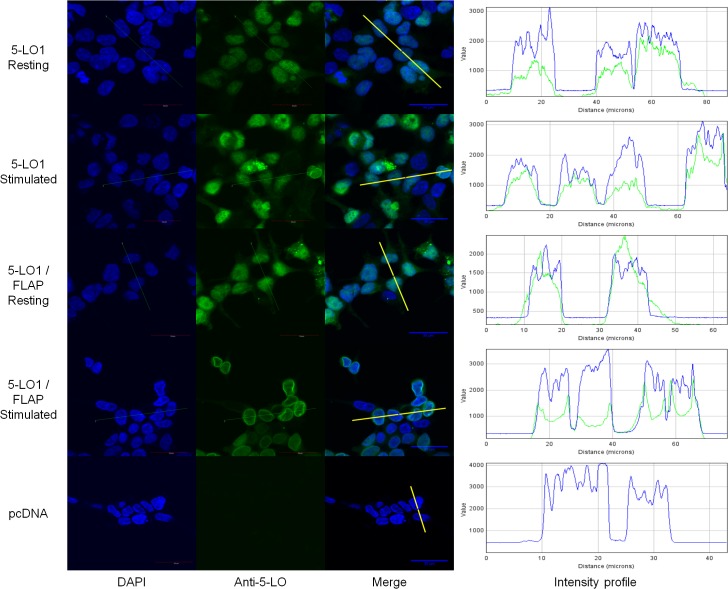
Sub-cellular localization of wild-type (WT) 5-lipoxygenase in cells expressing FLAP or not. HEK293 cells expressing FLAP or not were transfected with a vector expressing 5-LO1 or with a control vector (pcDNA). Cells were stimulated with 1 μM A23187 and 10 μM AA or were incubated with their diluent (resting) for 10 minutes. Cells were then fixed and permeabilized and then incubated with rabbit anti-5-LO. Slides were then incubated with an Alexa488-conjugated secondary anti rabbit antibody (green) and with DAPI (blue) to visualize nuclei, and slides were then mounted. Samples were analysed by confocal microscopy and images are presented on the left panels. The intensity of the signals could be visualized by intensity profiles (right panels) of the regions indicated by the white line on the merge images. Images are representative of three independent experiments. Scale bar represents 30 μm.

To further characterize the behaviour of the 5-LO isoforms, the intracellular localization of 5-LO1 and 5-LO∆13 were evaluated by confocal microscopy. 5-LO1 was primarily located in the nucleoplasm of resting HEK293 cells, whether the cells expressed FLAP or not (Fig 4). Upon cell stimulation, 5-LO1 translocation was observed only in cells co-expressing FLAP as shown by the strong peri-nuclear rings, confirming the importance of FLAP in 5-LO1 translocation to the nuclear envelope in these cells (Fig 4). In addition to the confocal microscopy images, the intracellular location of 5-LO can also be visualized graphically with intensity profiles of cross sections of cells (Fig 4, right panels). The intensity profiles show that 5-LO staining overlaps primarily with that of nuclear DNA staining (DAPI) in the nuclear compartment of resting cells, and spikes of 5-LO intensity appear at the periphery of DNA staining in stimulated FLAP-expressing cells, consistent with translocation to the nuclear membrane.

**Fig 5:**
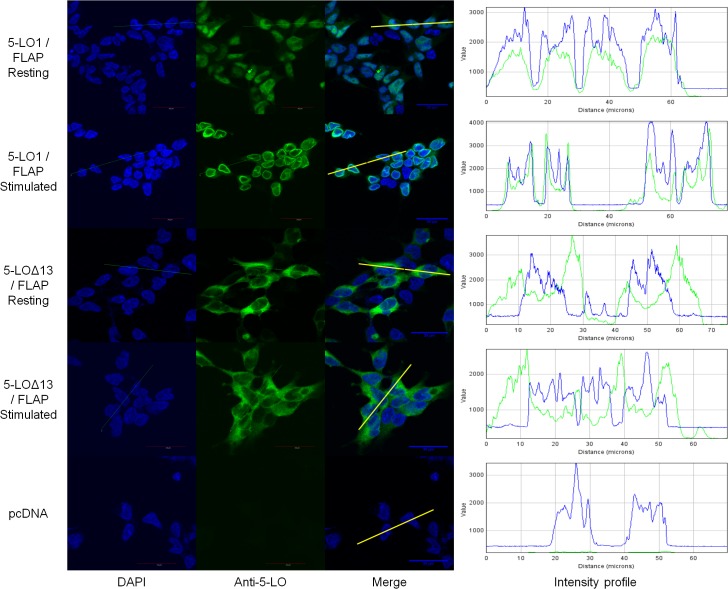
Sub-cellular localization of 5-LO1 and 5-LO∆13 in resting and stimulated cells. HEK293 cells expressing FLAP which were transfected to express either 5-LO1 or 5-LO∆13, or which were transfected with a control vector (pcDNA) were stimulated with 1 μM A23187 and 10 μM AA or were incubated with their diluent (resting) for 10 minutes. Cells were then fixed and permeabilized and then incubated with rabbit anti-5-LO. Slides were then incubated with an Alexa488-conjugated secondary anti-rabbit antibody (green) and with DAPI (blue) to visualize nuclei, and slides were then mounted. Samples were analysed by confocal microscopy and images are presented on the left panels. The intensity of the signals could be visualized by intensity profiles (right panels) of the regions indicated by the white line on the merge images. Images are representative of three independent experiments. Scale bar represents 30 μm.

Unlike the 5-LO1, 5-LO∆13 was primarily located in the cytoplasm of resting cells (Fig 5). Interestingly, no translocation of 5-LO∆13 was observed following cell stimulation in the presence of FLAP (Fig 5) or in its absence (not shown). Closer observation of cells using the intensity profiles of cross sections of cells revealed that the maximum of the intensity profile signal for 5-LO∆13 was at close proximity to the nuclear envelope, and that this signal tends to fade in more distal cytoplasmic regions (Fig 5, right panels). However, this small perinuclear accumulation is not as pronounced or sharp as that observed for translocation of 5-LO1 to nuclear membranes in stimulated cells.

**Fig 6:**
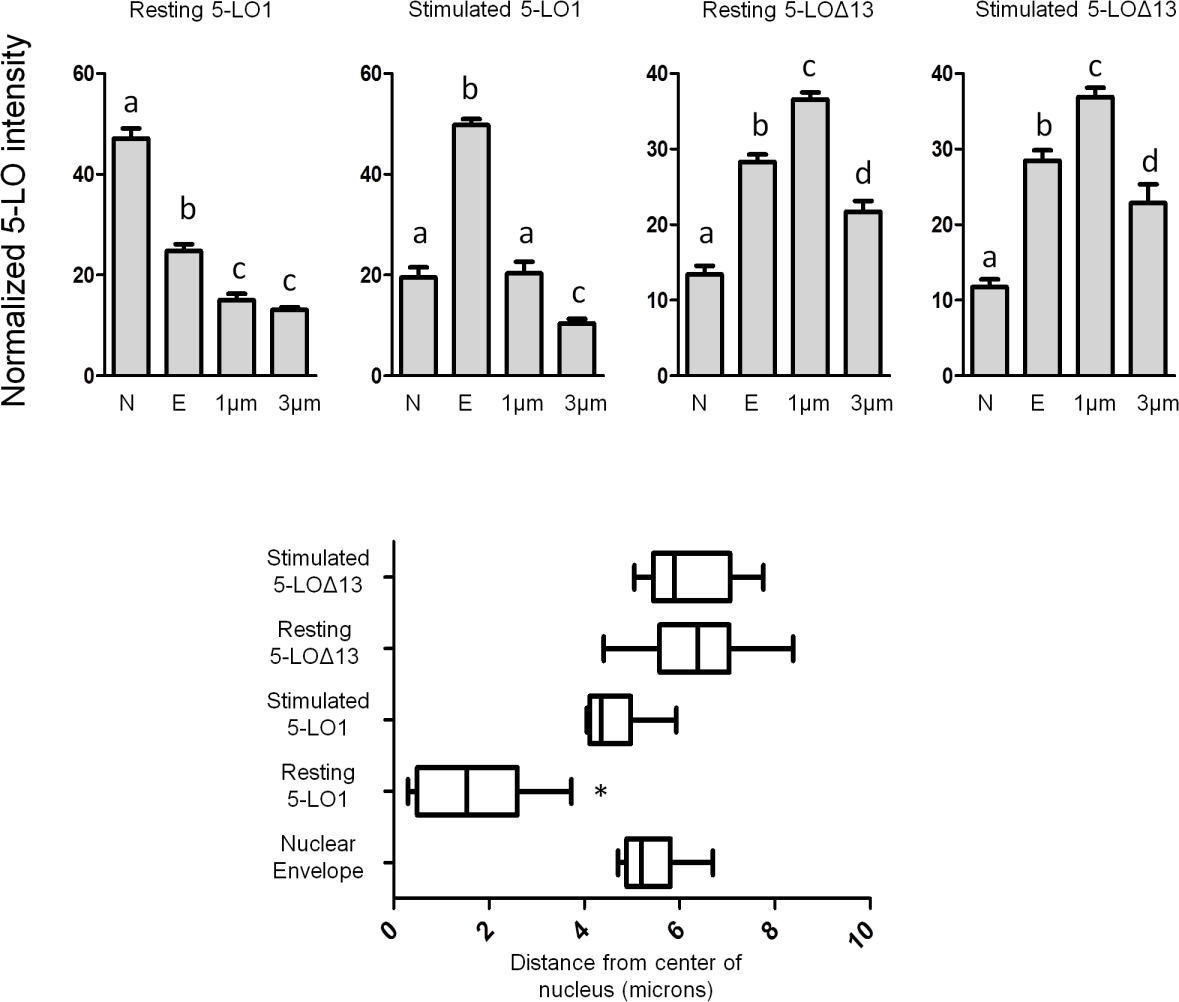
Intensity profiles of of 5-LO1 and 5-LO∆13 staining in resting and stimulated cells. HEK293 cells expressing FLAP which were transfected to express either 5-LO1 or 5-LO∆13 were stimulated with 1 μM A23187 and 10 μM AA or were incubated with their diluent (resting) for 10 minutes. Cells were then fixed and permeabilized and then incubated with rabbit anti-5-LO. Slides were then incubated with an Alexa488-conjugated secondary anti-rabbit antibody and with DAPI to visualize nuclei, and slides were then mounted. Samples were analysed by confocal microscopy and the intensity of the signals of cross-sections of cells were measured as in Fig 5. **Top panels:** The intensity of each cross section was measured at the centre of the nucleus (N), at the nuclear envelope (E) identified by the edge of DAPI staining, at 1 μm and at 3 μm from the nuclear envelope (cytoplasmic side). Values are means ± SEM, n = 9. Values in the same figure without a common superscript are different, p<0.05. **Bottom panel:** Box and whisker plots showing the distance from the centre of the nucleus of the most intense anti-5-LO staining of cross sections. The box represents the two middle quartiles of data, the vertical line in the box is the median and the whiskers represent the minimum and maximum of all the data. *Median value different from others, p<0.05. Values in all panels are from intensity cross sections of 9 cells per condition from three different fields of view, each field representing a separate experiment.

The ability to measure intensity profiles of cross-sections of cells was utilized to quantify the cellular distribution of 5-LO1 and 5-LO∆13 in resting and stimulated cells. The intensity of anti-5-LO staining was measured at the centre of the nucleus, at the nuclear envelope as determined by the edge of DAPI staining, 1 μm from the edge of the nucleus to represent perinuclear staining and at 3 μm from the nucleus to represent cytosolic staining (Fig 6, top panels). 5-LO1 was significantly more abundant in the nucleus in resting cells compared to other areas, while resting 5-LO∆13 staining was significantly greater in the perinuclear and nuclear envelope regions. Upon cell stimulation 5-LO1 became significantly more intense at the nuclear envelope region whereas the pattern of 5-LO∆13 staining was unchanged. When site of maximum staining intensity was determined on cell cross sections, resting 5-LO1 was significantly closer to the centre of the nucleus than stimulated 5-LO1 or 5-LO∆13 (resting or stimulated) whose maximum intensities were not significantly different from one another (Fig 6, bottom panel).

**Fig 7:**
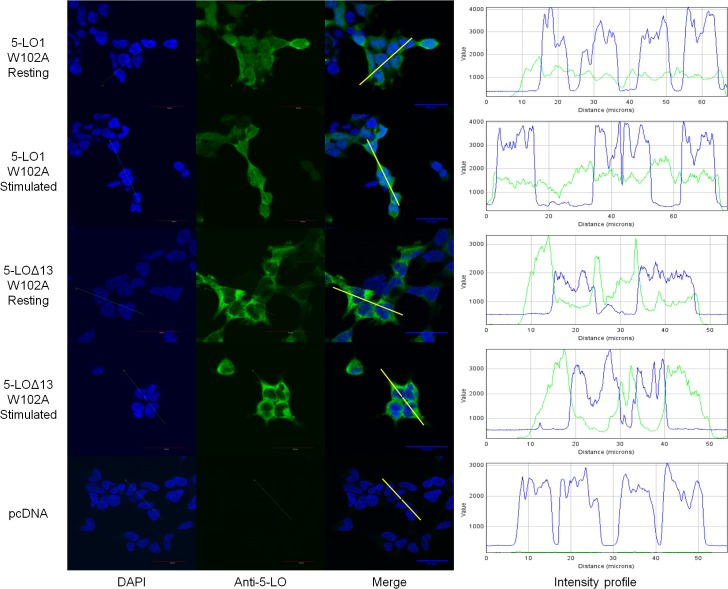
Sub-cellular localization of the W102A mutants of 5-LO1 and 5-LO∆13 in resting and stimulated cells. HEK293 cells expressing FLAP and expressing either the 5-LO1-W102A mutant or the 5-LO∆13-W102A mutant, or which were transfected with a control vector (pcDNA) were stimulated with 1 μM A23187 and 10 μM AA or were incubated with their diluent (resting) for 10 minutes. Cells were then fixed and permeabilized and then incubated with rabbit anti-5-LO. Slides were then incubated with an Alexa488-conjugated secondary anti-rabbit antibody (green) and with DAPI (blue) to visualize nuclei, and slides were then mounted. Samples were analysed by confocal microscopy and images are presented on the left panels. The intensity of the signals could be visualized by intensity profiles (right panels) of the regions indicated by the white line on the merge images. Images are representative of three independent experiments. Scale bar represents 30 μm.

Although 5-LO1 and CLP have been shown to co-associate in nuclear fractions of stimulated cells [17, 19] and 5-LO1 translocation is greatly diminished in the absence of CLP [18], the subcellular distribution of 5-LO1-W102A mutants had not been previously investigated. Fig 7 shows that the 5-LO1-W102A has a uniform cellular distribution in resting cells suggesting that W102 is required for the nuclear location of WT 5-LO. Stimulation of cells expressing 5-LO1-W102A did not induce 5-LO translocation to the nuclear envelope, consistent with its inability to generate 5-LO products. 5-LO∆13-W102A mutants displayed a cellular distribution profile similar to that of 5-LO∆13 with the increased staining in the perinuclear region and no difference between stimulated and non-stimulated cells (Fig 7).

### 5-LO isoforms have distinct phosphorylation states

**Fig 8:**
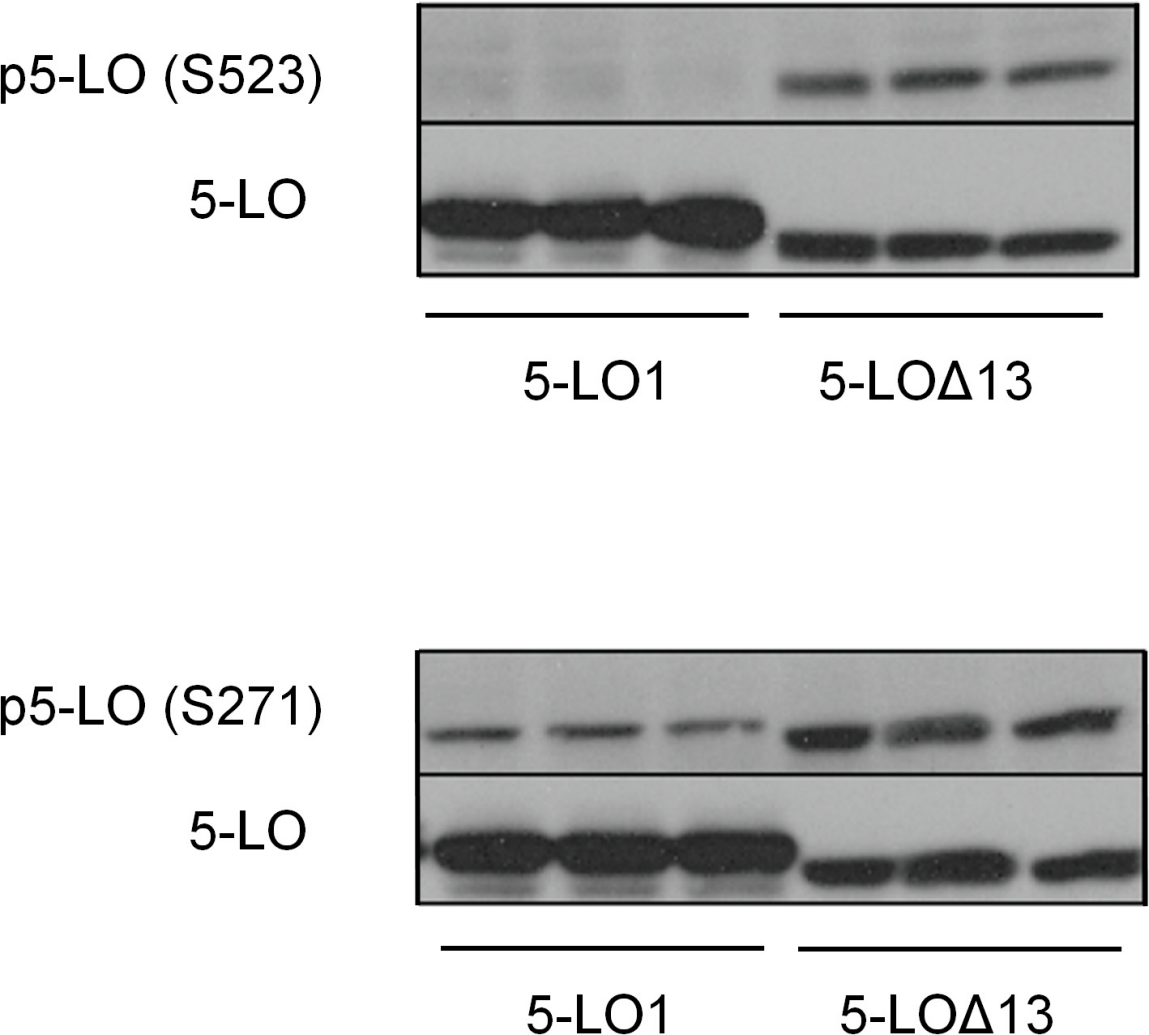
Phosphorylation pattern of 5-LO1 and 5-LO∆13 is different in HEK293 cells. Immunoblot analysis of resting HEK293 cells expressing either 5-LO∆13 or 5-LO∆13. All cells also expressed FLAP and CLP. After separation of proteins by SDS-PAGE, membranes were subject to western blots using anti phospho-serine 523 (S523) or anti-phospho-serine 271 (S271). Expression of 5-LO isoforms was verified by blotting using anti-5-LO (5-LO). The immunoblots show the results of 3 independent experiments.

Given the distinct sub-cellular distribution of 5-LO1 and 5-LO∆13, the phosphorylation patterns of these two isoforms were investigated. Western blotting was performed on extracts from stable transfectants using specific antibodies against phosphorylated S-523 and S-271. Despite the fact that both protein isoforms are identical other than the absence of amino acids 559–615 in 5-LO∆13, 5-LO∆13 was phosphorylated on S523 while 5-LO1 was not (Fig 8). Similarly, S271 was heavily phosphorylated on 5-LO∆13 while 5-LO1 was only moderately phosphorylated.

## Discussion

5-LO is subjected to a number of regulatory mechanisms that impact on the capacity to produce leukotrienes. Recently a potentially new mechanism of 5-LO regulation was revealed when several catalytically inactive splice variants of human 5-LO were identified [26, 27, 36] and shown to negatively impact on the biosynthesis of 5-LO products when co-expressed with the active 5-LO1. Human polymorphonuclear leukocytes (PMN) and several human myeloid and lymphoid cell lines were shown to express alternatively-spliced 5-LO transcripts, and western blots from human PMN and Raji lymphoma cells showed immunoreactive bands that co-migrated with 5-LO∆13 [26].

In the current study, further investigation of the 5-LO∆13 protein isoform provides new insight into the control of 5-LO1 activation and leukotriene biosynthesis. The relationship between 5-LO∆13 expression and 5-LO product biosynthesis was expression-dependent where increased expression of 5-LO∆13 resulted in a greater inhibition of 5-LO product biosynthesis (Fig 2). Surprisingly, in both HEK293 and HeLa cells 5-LO∆13 mainly inhibited cellular LT biosynthesis with a less pronounced impact on 5-HETE production. This was akin to the effect of CLP on recombinant 5-LO1 activity where CLP enhances LT synthesis *in vitro* but has little influence on 5-HETE synthesis [17, 19]. Likewise, FLAP silencing in MM6 cells was also recently associated with a decrease in LT biosynthesis but not that of 5-HETE [18]. Therefore, this inhibition profile was suggestive of an antagonist-like effect of 5-LO∆13 where this isoform may interfere with the ability of 5-LO1 to interact with CLP and/or FLAP.

This observation prompted the investigation of the potential implication of CLP or FLAP in the inhibitory effect of 5-LO∆13. Previous reports had shown that CLP enhances recombinant 5-LO1 activity in cell-free systems, while CLP silencing experiments recently confirmed a role for CLP in 5-LO1 activation in intact cells [18]. W102 is the key 5-LO1 residue required for the activity-enhancing interaction of 5-LO1 with CLP in cell free assays [17, 19], however the importance of W102 on 5-LO1 activation in stimulated whole cells had never been evaluated. The current study shows that the stimulation of HEK293 cells expressing the W102A mutant of 5-LO1 resulted in very little biosynthesis of 5-LO products (Fig 3). Having confirmed the requirement of W102 in intact cells, the interaction of 5-LO∆13 with CLP was investigated using 5-LO∆13-W102A mutants that should also be unable to interact with CLP. However, the 5-LO∆13-W102A mutants showed the same inhibitory capacity as the non-mutated form of the protein (Fig 3) suggesting that the mechanism by which the 5-LO∆13 inhibits 5-LO1 does not involve an interaction or competition with CLP. Similarly, although the absence of FLAP decreased the cellular capacity to synthesize 5-LO products, 5-LO∆13 was fully capable of inhibiting 5-LO product biosynthesis in stimulated HEK293 cells in the absence of FLAP indicating that the inhibitory effect of 5-LO∆13 does not involve FLAP.

The control of the subcellular location of 5-LO1 and its ability to translocate to nuclear membranes following cell stimulation are also mechanisms involved in the regulation of 5-LO product biosynthesis. Surprisingly, the location of 5-LO1, 5-LO∆13 and their W102A mutants varied greatly in resting HEK293 cells (Figs 5 and 6). Similar to previous studies using HEK293 cells as well as human alveolar macrophages, mast cells and adherent neutrophils and eosinophils [9, 11, 37, 38], 5-LO1 was primarily located in the nucleoplasm of resting HEK293 cells although some cytoplasmic 5-LO was also evident. 5-LO1 protein also translocated to nuclear membranes following cell stimulation in a FLAP-dependent manner, as previously shown in other cell types [13, 18]. Unexpectedly, expression of 5-LO1-W102A mutants revealed a significant shift in the protein’s localization in resting cells showing a diffuse cellular staining as well as a complete loss of the ability to translocate following stimulation (Fig 7), consistent with the inability to activate this mutant following cell stimulation. It was recently shown that CLP silencing results in a loss in the ability of 5-LO to translocate to the nucleus in stimulated MM6 cells. However, in the present study the loss of nuclear localization in resting HEK293 cells indicates that W102 is important for nuclear localisation of 5-LO1 and suggests that interaction with CLP may be required for nuclear targeting. Thus the role of CLP may be more complex than previously thought.

The cytosolic location of 5-LO∆13 differed considerably from that of the 5-LO1 protein. The intensity profiles obtained from microscopy images show that 5-LO∆13 is primarily non-nuclear and appears to be concentrated in the ER-rich peri-nuclear region. However, this subcellular location was not altered by cell stimulation or by expression of the 5-LO∆13-W102A mutant (Figs 5–7). This peri-nuclear position of 5-LO∆13 is the location in HEK293 cells where both proteins isoforms are in proximity to one another following cell stimulation and suggests that this is where 5-LO∆13 may interact with 5-LO1 and exert its inhibitory effect. Since 5-LO forms dimers that might be required for its activation [22–24], this inhibitory effect could possibly occur through the formation of inactive heterodimers, or indirectly through another unknown mechanism. Additionally, the altered location of 5-LO∆13 compared to 5-LO1 suggests that the presence of amino acids 559 through 615 of 5-LO1 that are coded by exon 13 are required for both nuclear import and stimulus-induced translocation. Accordingly, it was previously suggested that mutations that eliminate enzymatic activity also prevent nuclear localization of 5-LO by a yet-to-be-described mechanism implying that enzymatic activity is required for nuclear import [39]. Interestingly, splice variants of the human 15-lipoxygenase 2, the most abundant AA-metabolizing enzyme in prostate cancer, are also catalytically inactive, cytosolic proteins as opposed to their nuclear, enzymatically-active counterpart [40]. Therefore, the existence of alternatively spliced lipoxygenase isoforms that do not co-localize with their enzymatically active counterparts is not limited to 5-LO.

The known nuclear localisation sequences and phosphorylation sites associated with the control of the subcellular location of 5-LO are all retained in the Δ-13 isoform (Fig 1). However 5-LO1 and 5-LO∆13 show strikingly dissimilar phosphorylation patterns on S523 and S271 where 5-LO∆13 is hyper-phosphorylated (Fig 8). This phosphorylation pattern may explain the differential localization of these proteins since phospho-S523, that lies within the BR^518^ import region and is targeted by protein kinase A, prevents nuclear import [31] thus preventing 5-LO∆13 entry into the nucleus. On the other hand, 5-LO1 that enters the nucleus is retained due to the phosphorylation of Ser-271, which prevents nuclear export of 5-LO1 [30]. Therefore, the differential location of the 5-LO protein isoforms may be the consequence of different susceptibilities of the isoforms to protein kinases or phosphatases.

Overall this study provides new information regarding inhibition of LT biosynthesis by 5-LO∆13, as well as the role of the sequence coded by exon 13, the W102 residue of 5-LO1, and possibly that of CLP, on nuclear targeting of 5-LO. Given that alternatively spliced isoforms of 5-LO have been identified in human leukocytes, a better understanding of the regulation of their expression and of the mechanisms by which they modulate LT biosynthesis will contribute to our understanding of the complex regulation of LT biosynthesis and may possibly lead to new approaches to the treatment of diseases like asthma and atherosclerosis in which 5-LO plays an active role.
